# Paired Validation of a Multimodal Vision-Transformer System for the Non-Invasive Diagnosis of Idiopathic Pulmonary Fibrosis

**DOI:** 10.3390/diagnostics16152398

**Published:** 2026-07-30

**Authors:** Omid Tahamtani-Omran, Angad Kalra, Michael Muelly, Joshua Reicher, Roaa Ahmed

**Affiliations:** 1 Department of Chemistry and Biochemistry, University of California, Los Angeles, 607 Charles E. Young Drive East, Los Angeles, CA 90095, USA; omid.tahamtani.o@gmail.com; 2 IMVARIA Inc., 2930 Domingo Ave #1496, Berkeley, CA 94705, USA; 3 School of Medicine, Ahfad University for Women, P.O. Box 167, Omdurman 14411, Sudan

**Keywords:** idiopathic pulmonary fibrosis, interstitial lung disease, deep learning, vision transformer, computed tomography, predetermined change control plan

## Abstract

**Background/Objectives:** Distinguishing idiopathic pulmonary fibrosis (IPF) from other interstitial lung diseases is prognostically and therapeutically important. We developed Fibresolve, a machine learning system that analyzes chest computed tomography (CT) to support IPF diagnosis. Fibresolve version 1 (v1) was validated in the PUFAIR study, met its co-primary endpoints, and received U.S. FDA authorization. Fibresolve version 2 (v2) was subsequently developed under the FDA’s Predetermined Change Control Plan (PCCP) framework and, here, is evaluated against the authorized v1 model. **Methods:** v2 augments the original CT-only v1 classifier by incorporating age, sex, and forced vital capacity into an ensemble, while retaining the v1 CT-only model as a fallback when these variables are unavailable. Both models were evaluated in the same 300-patient PUFAIR cohort (83 IPF, 217 non-IPF) from two U.S. centers. Reference-standard diagnoses were established by institutional multidisciplinary discussion and supported by surgical pathology in 94.7% of cases. McNemar test, Cohen κ, and bootstrap noninferiority analyses were used for paired model comparisons. **Results:** In the key thin-slice diagnostic CT subgroup (N = 137), v2 achieved a sensitivity of 57.5% (95% CI, 42.2–71.5) and specificity of 84.5% (95% CI, 76.0–90.4), compared with 55.0% (95% CI, 39.8–69.3) and 82.5% (95% CI, 73.7–88.8) for v1. Performance improvements were directionally consistent across most subgroups, with no evidence of inferior performance. **Conclusions:** Fibresolve v2 preserved the performance of the FDA-authorized v1 model while providing modest, directionally favorable improvements. These findings support v2 as the primary model, with v1 retained as a robust fallback when clinical variables are unavailable.

## 1. Introduction

The non-invasive diagnosis of idiopathic pulmonary fibrosis (IPF), and its distinction from the other 200-plus interstitial lung diseases (ILDs), is important for prognosis and treatment yet remains subjective and dependent on subspecialty expertise. Fibresolve (IMVARIA Inc., Berkeley, CA, USA) is the first machine-learning system authorized by the U.S. Food and Drug Administration (FDA) to aid this diagnosis, having received De Novo marketing authorization (DEN220040) as an adjunct in the diagnosis of IPF prior to invasive testing [1]. Analyzing the complete chest computed tomography (CT) volume in three dimensions, it learns a broad set of imaging correlates of IPF and can indicate the diagnosis even in cases that lack a classic usual interstitial pneumonia (UIP) appearance [2]. In the pivotal PUFAIR study [3], Fibresolve version 1 (v1) met both of its pre-specified co-primary endpoints, contributing to the technology’s FDA authorization in 2024.

A central challenge for AI-enabled diagnostics is that their algorithms are expected to improve iteratively over time, yet significant modifications have traditionally required separate regulatory submissions, lengthening timelines for making such improvements available for clinical use. The FDA’s Predetermined Change Control Plan (PCCP) framework was finalized in December 2024 to address this challenge: the framework permits pre-specified modifications to an authorized device to be implemented and validated against the originally authorized version without a new marketing submission, provided each change remains within an agreed scope [4]. The present study reviews the application of this framework to the Fibresolve technology, and assesses the resulting performance changes. We advance the software from version 1 to version 2 (v2), which augments the original CT-only classifier with a vision-transformer model incorporating age, sex, and forced vital capacity (FVC) in an ensemble, while retaining the v1 CT-only model as a fallback when these ancillary variables are unavailable. In alignment with the PCCP framework, version 2 is then validated head-to-head against the originally authorized version 1.

To briefly review the state of the disease: IPF is the most common idiopathic interstitial pneumonia, and although historically a progressive disease with a poor prognosis, it now follows a more favorable course with antifibrotic therapy [5,6,7]. Effective management depends on distinguishing IPF from the other ILDs [8,9]. This distinction can be made non-invasively when a UIP pattern on CT is accompanied by compatible clinical findings, but in practice it is subjective and only moderately reproducible even among experts (weighted κ≈ 0.48–0.65) [10,11]. When the imaging is inconclusive, a surgical lung biopsy may be required but carries appreciable morbidity and mortality [12], and access to the subspecialty expertise that these judgments demand is often delayed [13,14]. These limitations motivated the development of objective, automated tools, and machine-learning analysis of chest CT in particular has been shown to identify fibrosis-related features beyond standard imaging criteria and to correlate with histopathology and long-term outcomes in fibrotic lung disease [15,16,17,18,19]. These clinical challenges drove the development of Fibresolve v1 and contributed to methodologies for improvement for v2.

Two developments in machine learning motivated the v2 update. First, transformer architectures, originally introduced for sequence modeling [20] and subsequently adapted to images as the Vision Transformer (ViT) [21], have become a leading approach in medical image analysis, where global self-attention (i.e., the ability to gather more holistic image context) can capture the diffuse, spatially distributed patterns that characterize fibrotic lung disease [22]. Second, multimodal models that combine imaging with structured clinical data may be anticipated to outperform imaging-only models for disease classification and prognosis [23]. FVC in particular is a validated and clinically meaningful measure in IPF and is therefore a natural candidate input [24,25]. Reflecting this, subsequent work has demonstrated that a multimodal Fibresolve classifier also predicts mortality across ILDs [26], complementing CT-based prognostic findings reported in independent registry cohorts [27,28]. The ongoing evolution of datasets, machine learning architecture, and addition of multimodal inputs all contribute to potential algorithm performance improvements.

To validate this update under the PCCP with maximal internal validity, we re-analyzed the identical 300-patient PUFAIR cohort with both versions and compared their performance using paired statistical methods. The purpose of this study is: (1) to describe the version 2 architecture and its fallback design; (2) to compare the diagnostic performance of version 2 with version 1 across the full dataset and the key thin-slice diagnostic subgroup, following the analysis structure of the original PUFAIR study; and (3) to formally assess the non-inferiority of version 2 relative to version 1.

## 2. Materials and Methods

### 2.1. Study Design and Cohort

This work is a paired cohort comparison of the two Fibresolve versions applied to one locked dataset, namely the cohort assembled for PUFAIR, whose assembly and design we summarize here and report in full elsewhere [3]. In brief, PUFAIR adopted a retrospective–prospective design (per National Cancer Institute definitions), in which prospectively accrued cases were re-scored with outcomes withheld from the model developers. The source material was drawn from a large multinational collection of registries and completed clinical trials accrued between 2005 and 2018, from which two US sites contributed the analyzed cases. Eligibility required an age above 18 years, a complete symptom history, and a CT examination with contiguous 1–5 mm axial sections covering the lungs in full; 300 of 369 candidate cases met these criteria and the target sample size. Because both software versions were evaluated on this same fixed cohort, its baseline demographic, clinical, and technical characteristics are identical to those of PUFAIR and are reproduced in Appendix A for reference (Table A1 and Table A2).

The reference diagnosis was first assigned by the local team and verified by the coordinating-site investigator, and the definitive label was then established through a multidisciplinary discussion informed by surgical pathology and longitudinal follow-up. Surgical tissue was available for 94.7% of patients. Both Fibresolve versions operated on data available at the baseline (time-zero) assessment, and their binary outputs were compared against the ground truth to derive performance measures.

### 2.2. Predetermined Change Control Plan

The diagnostic update to Fibresolve was implemented under a Predetermined Change Control Plan (PCCP), a regulatory mechanism authorized by the FDA as part of the device’s original marketing authorization (De Novo DEN220040) that permits prospectively specified modifications to a locked machine learning device to be made without a new premarket submission, provided they are executed within an agreed-upon framework. A PCCP comprises two core components: the SaMD Pre-Specifications (SPS), which define the specific types of modification that may be made to the device, and the Algorithm Change Protocol (ACP), which defines the methods used to develop, verify, validate, and control each modification so that the device remains safe and effective. The ACP follows the structure described in the FDA’s guidance on marketing-submission recommendations for PCCPs [4] and adheres to Good Machine Learning Practice, organizing the change process across data management, model retraining, performance evaluation, and update procedures. Consistent with the protocol, the updated device was required to meet the same pre-specified statistical endpoints for overall sensitivity and specificity established for the original device, with retraining, tuning, and locked test-set evaluation conducted on data partitioned to prevent any patient-level overlap, and with subgroup performance assessed against the prior version to confirm non-inferiority before deployment.

### 2.3. Fibresolve Version 1

Fibresolve is a cloud-deployed pipeline that ingests CT data and applies a machine-learning classifier to predict whether a case will ultimately be diagnosed as IPF, taking the final clinical diagnosis established with surgical biopsy and/or follow-up as the reference [2]. Rather than confining itself to the canonical hallmarks of UIP, the model learns a wider set of imaging correlates of IPF, which allows it to flag cases that do not present a classic UIP appearance. It was trained on a development population that exceeded 2000 patients, processing each complete CT scan in three dimensions; at its locked, pre-specified threshold, it achieved a sensitivity of 67% and a specificity of 90% on the development validation data, as previously reported [2]. In version 1, the classifier draws on the CT images alone and does not use any clinical, demographic, or laboratory information.

### 2.4. Fibresolve Version 2

**Figure 1:**
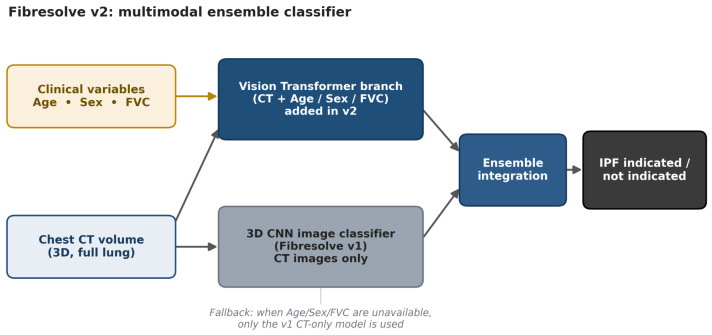
Overview of the Fibresolve version 2 architecture. The chest CT volume is analyzed by both the original three-dimensional convolutional image classifier (version 1) and a new vision-transformer branch that additionally ingests age, sex, and FVC. The two branches are combined in an ensemble to produce the binary IPF indication. When age, sex, or FVC are unavailable, the system falls back to the version 1 CT-only model. Abbreviations: CT, computed tomography; 3D, three-dimensional; CNN, convolutional neural network; ViT, vision transformer; FVC, forced vital capacity; IPF, idiopathic pulmonary fibrosis; v1, Fibresolve version 1; v2, Fibresolve version 2.

The version 2 (v2) Fibresolve classifier was developed by augmenting the original deep residual convolutional neural network (CNN), a 3D ResNet-101 ensemble that discriminates idiopathic pulmonary fibrosis (IPF) from other interstitial lung diseases on thoracic CT, with a new multimodal vision transformer (ViT), the two components combined as an ensemble to generate the final diagnostic output (Figure 1). A vision transformer is a deep learning architecture that, rather than relying on the localized filters of a CNN, partitions an image into a sequence of patches and applies self-attention to weigh the relationships among all regions of the image simultaneously, allowing each local finding to be interpreted within the context of the entire scan—it essentially improves on the contextualization of features within the images. The underlying architecture is analogous to the transformers that power large language models but adapted to the analysis of imaging features rather than language. The component added in v2 is a multimodal ViT, meaning the architecture was extended beyond imaging to accept additional input modalities—continuous numeric variables (forced vital capacity and age) and a binary variable (sex)—using multimodal attention (fusion) layers that learn to weight and integrate these heterogeneous inputs alongside the CT data. The multimodal ViT uses a standard patch-based self-attention architecture adapted for CT volumes, with clinical variables (age, sex, FVC) incorporated via fusion layers prior to the final classification head. The CNN and ViT components were combined at the ensemble level rather than trained jointly. Full architectural specifications, training procedure, and preprocessing pipeline are proprietary but follow standard training protocols for similar models. This multimodal ViT was trained on the same multi-institution U.S. development cohort used to train v1—a population distinct from, and with no case-level overlap with, the locked PUFAIR cohort used for evaluation in this study—with the ancillary clinical inputs available in 63% of training cases, and was then combined with the v1 CNN to form the final v2 ensemble. Since FVC was not available in 100% of patients, the v1 CNN is retained as a fallback for cases in which ancillary data are absent and only CT images are available.

### 2.5. Statistical Analysis

Performance metrics for the diagnostic models, including sensitivity, specificity, positive predictive value (PPV), and negative predictive value (NPV), were calculated with 95% confidence intervals (CIs) using the Wilson score method. Diagnostic accuracy was further evaluated using likelihood ratios and odds ratios, each reported with 95% confidence intervals. Confidence intervals for likelihood ratios were calculated using the log-transformed method of Simel et al. [29]; confidence intervals for diagnostic odds ratios were calculated using the log-transformed Woolf method, with the Haldane–Anscombe correction applied when any cell count was zero. To compare the categorical predictions of the new model against the established reference, McNemar’s exact test was utilized for paired nominal data, supplemented by Cohen’s Kappa to assess inter-rater agreement. Statistical significance for model performance against pre-defined clinical thresholds (Sensitivity > 30%, Specificity > 80%) was determined via one-sided binomial tests. Furthermore, a non-inferiority analysis was conducted to compare the new model to the previous iteration using a −10% margin; the mean differences and associated 95% CIs were derived from a paired bootstrap resampling procedure with 3000 iterations. The −10% non-inferiority margin was pre-specified in the Algorithm Change Protocol, anchored to the lower CI bound of v1’s demonstrated performance; all non-inferiority tests were one-sided (α=0.05). Subgroup analyses were performed across demographic and clinical strata, including age, sex, and lung function (FVC), to ensure robust performance across diverse patient populations. All statistical computations were performed in Python using Pandas (v2.2.2), NumPy (v2.0.2), and SciPy (v1.16.3).

## 3. Results

As both Fibresolve versions were evaluated on the same fixed cohort assembled for PUFAIR, its baseline demographic, clinical, and technical characteristics are identical to those reported previously [3]. The 300 patients had a median age of 62 years and an approximately even sex distribution (49.7% female); the cohort was predominantly White (85.7%) and non-Hispanic (88.7%), and 69.0% were ever-smokers. Percent-predicted forced vital capacity (ppFVC) was recorded in 271 patients and ranged widely, from preserved (above 75% in 34.7%) to substantially reduced (below 50% in 19.7%). Imaging originated from scanners spanning four manufacturers and 23 distinct models, with slice thickness ranging from 1 to 5 mm, and surgical pathology was available in 94.7% of cases (284/300). Under the reference standard, IPF accounted for 27.7% (83/300) of final diagnoses; the most common non-IPF diagnoses were unclassifiable ILD (18.0%), chronic hypersensitivity pneumonitis (16.7%), and nonspecific interstitial pneumonia (10.7%), with the remainder distributed across numerous less common ILD subtypes.

**Figure 2:**
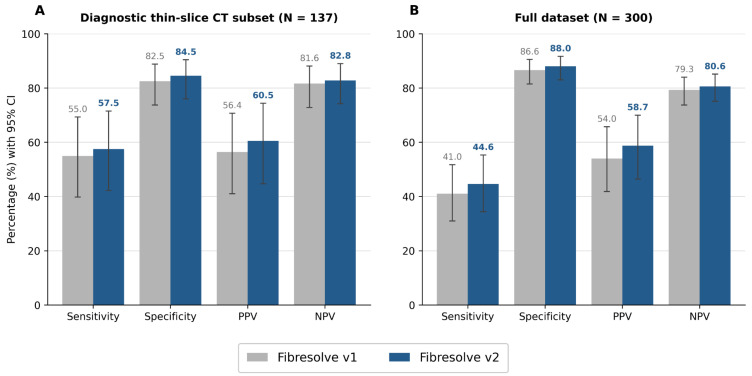
Diagnostic performance of Fibresolve v2 versus v1, shown for (**A**) the diagnostic thin-slice CT subset (N = 137) and (**B**) the full dataset (N = 300). Bars show point estimates with 95% confidence intervals. v2 point estimates equal or exceed v1 across all metrics in both cohorts. Abbreviations: CI, confidence interval; CT, computed tomography; PPV, positive predictive value; NPV, negative predictive value; v1, Fibresolve version 1; v2, Fibresolve version 2.

**Figure 3:**
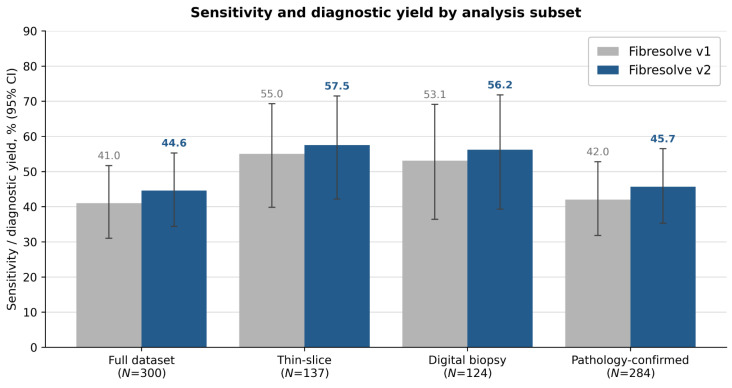
Sensitivity and diagnostic yield of Fibresolve v2 versus v1 across the full dataset, the diagnostic thin-slice subset, the indeterminate (“digital biopsy”) subgroup, and pathology-confirmed cases. Bars show point estimates with 95% confidence intervals; v2 equals or exceeds v1 in every subgroup shown. Abbreviations: CI, confidence interval; v1, Fibresolve version 1; v2, Fibresolve version 2.

The analysis of greatest clinical relevance is the thin-slice diagnostic CT subset (N = 137; 40 IPF, 97 non-IPF), which reflects the diagnostic-quality imaging on which Fibresolve is intended to be used. In this subgroup, Fibresolve v2 achieved a sensitivity of 57.5% (95% CI 42.2–71.5) and a specificity of 84.5% (76.0–90.4), compared with 55.0% (39.8–69.3) and 82.5% (73.7–88.8) for v1 (Table 1, Figure 2A). PPV rose from 56.4% to 60.5% and the diagnostic odds ratio from 5.75 (95% CI 2.55–12.98) to 7.40 (95% CI 3.21–17.03), with substantially overlapping confidence intervals indicating this difference should not be interpreted as statistically distinguishable. Agreement between versions was high (94.9%, κ = 0.87; McNemar *p* = 1.00). The clinical value is sharpest in the indeterminate subset of these cases, those not confirmed as IPF non-invasively and otherwise considered for biopsy (N = 124), where v2 delivered a non-invasive diagnostic yield of 56.2% (39.3–71.8) at 87.0% specificity (Table 2, Figure 3), exceeding the v1 yield of 53.1% and comparing favorably with the 36.1% (95% CI 33.4–38.9%) yield reported for transbronchial biopsy [8].

Across the full dataset (N = 300), which additionally includes non-dedicated and thick-slice acquisitions and therefore represents a harder test, v2 retained its advantage: sensitivity was 44.6% (34.4–55.3) versus 41.0% (31.0–51.7) and specificity 88.0% (83.0–91.7) versus 86.6% (81.5–90.5), with PPV improving from 54.0% to 58.7%, NPV from 79.3% to 80.6%, and the diagnostic odds ratio from 4.50 to 5.91 (Table 3, Figure 2B). The two versions agreed on 96.7% of cases (κ = 0.90) and the McNemar test was not significant (exact *p* = 1.00). Confidence intervals for the likelihood ratios and diagnostic odds ratios overlapped substantially between versions in all three analyses, consistent with the non-significant paired comparisons.

In pre-specified non-inferiority testing against a −10% margin, v2 was non-inferior to v1 for both endpoints. The paired difference in sensitivity (v2 − v1) was +3.6 percentage points (95% CI −1.2 to +9.1) and the paired difference in specificity was +1.4 points (95% CI −0.5 to +3.7); the non-inferiority null hypothesis was rejected for both (*p* < 0.001). Thus, v2 met the non-inferiority criterion while showing full-dataset point estimates that were all equal to or better than those of v1.

Because the ancillary clinical inputs required by v2 were not available for every patient, performance was additionally examined separately for the two pathways. FVC was available in 271 of 300 patients (90.3%), who were therefore evaluated by the v2 ensemble; the remaining 29 (9.7%) lacked FVC and were routed to the v1 CT-only fallback. Among the 271 v2-pathway cases (75 IPF, 196 non-IPF), v2 achieved a sensitivity of 42.7% (32/75; 95% CI 32.1–53.9) and a specificity of 87.8% (172/196; 95% CI 82.4–91.6). Among the 29 fallback cases (8 IPF, 21 non-IPF), the CT-only pathway achieved a sensitivity of 62.5% (5/8; 95% CI 30.6–86.3) and a specificity of 90.5% (19/21; 95% CI 71.1–97.3). In the thin-slice subset (N = 137), FVC was available in 121 patients (88.3%); the v2 pathway (36 IPF, 85 non-IPF) achieved 55.6% sensitivity (20/36; 95% CI 39.6–70.5) and 84.7% specificity (72/85; 95% CI 75.6–90.8), and the 16 fallback cases (4 IPF, 12 non-IPF) yielded 75.0% sensitivity (3/4; 95% CI 30.1–95.4) and 83.3% specificity (10/12; 95% CI 55.2–95.3). The fallback strata contained only eight and four IPF-positive cases, respectively; their estimates are correspondingly imprecise and are reported to characterize pathway usage rather than to support a formal pathway comparison. Within these limits, the fallback pathway showed no evidence of degraded performance relative to the v2 pathway.

**Figure 4:**
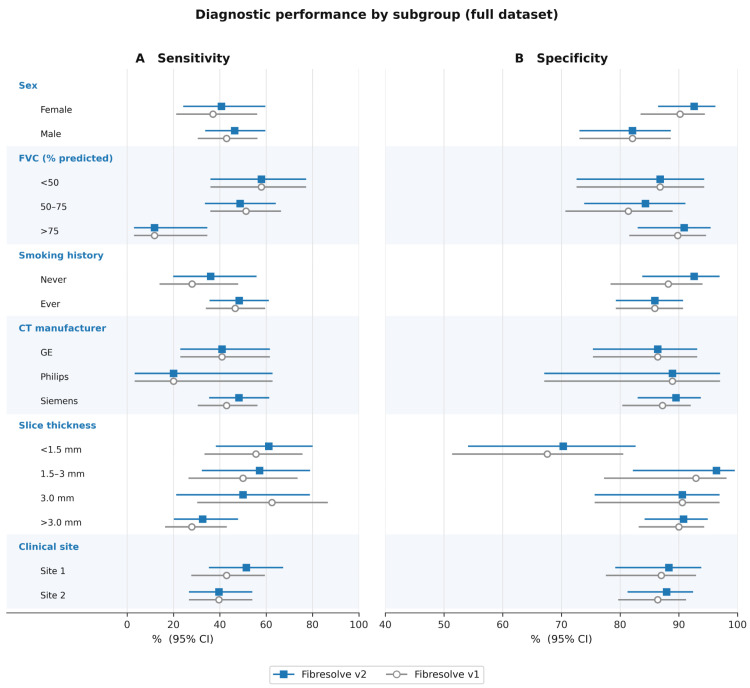
Diagnostic performance by subgroup (full dataset). Sensitivity (panel (**A**)) and specificity (panel (**B**)) for Fibresolve v1 (grey circles) and v2 (blue squares), each with 95% confidence intervals, across pre-specified demographic, clinical, and technical subgroups. Confidence intervals overlap substantially between versions within every stratum. Given the number of comparisons and lack of adjustment for multiplicity, no individual subgroup difference should be interpreted as a reliable effect. Abbreviations: CI, confidence interval; CT, computed tomography; FVC, forced vital capacity; IPF, idiopathic pulmonary fibrosis; v1, Fibresolve version 1; v2, Fibresolve version 2.

Performance was examined across demographic, clinical, and technical subgroups (Figure 4). Subgroup-level differences in both sensitivity and specificity were generally small relative to their confidence intervals and are not adjusted for multiple comparisons. These analyses are exploratory and intended only to assess for gross inconsistency across acquisition and demographic strata, not to support subgroup-specific claims. Performance was otherwise broadly consistent across CT manufacturers, clinical sites, and slice-thickness groups, supporting generalizability across acquisition settings. As anticipated, sensitivity was lowest in patients with preserved lung function (ppFVC > 75%), where it was 11.8% for both versions, indicating that the multimodal inputs did not improve detection of the milder or earlier disease that is most difficult to identify from imaging; both versions performed best in cases with reduced FVC and on thinner-slice acquisitions.

Since prior ILD literature commonly excludes certain diagnoses, performance was re-estimated under sequential exclusion of UILD, CTD-ILD, and CHP cases (Table 4). Sensitivity was unchanged by these exclusions for both versions (44.6% v2, 41.0% v1 in the full dataset), as expected, since the IPF-positive cases are unaffected. Specificity was stable or improved, most notably when UILD cases were excluded; differences between the versions were small and in both directions across the exclusion scenarios, with version 2 modestly higher in most and marginally lower in the most stringent (e.g., 88.0% vs 89.0% when all three categories were excluded from the full dataset). Results in the thin-slice subgroup were consistent.

## 4. Discussion

This study evaluated Fibresolve v2, an algorithmic update to Fibresolve v1, the first FDA-authorized AI-enabled tool for supporting the diagnosis of IPF [1]. The update was implemented under the device’s Predetermined Change Control Plan (PCCP), the regulatory framework that permits pre-specified modifications to an authorized device to be implemented and validated head-to-head against the originally authorized version, provided each change remains within an agreed scope and preserves safety and effectiveness [4]. Rather than retraining the model freely, we made a single pre-specified modification, a multimodal vision-transformer branch incorporating age, sex, and FVC, and validated it against the deployed version on the locked PUFAIR cohort [3].

In this paired re-analysis, Fibresolve v2 matched or exceeded v1 across the full dataset and both diagnostic subsets and was non-inferior to v1 for both sensitivity and specificity against the pre-specified margin, while preserving high agreement (κ = 0.87–0.90; case-level concordance 94.9–96.7%). The update maintained the established behavior of the deployed model while improving classification in a small number of borderline cases. Although v2 consistently matched or exceeded v1 across the overall cohort and most subgroups, the magnitude of improvement was modest and the paired comparisons were not statistically significant.

The principal change in v2 was the addition of a vision-transformer branch that integrates age, sex, and FVC alongside CT imaging. On the full dataset, v2 identified additional true IPF cases while simultaneously reducing false-positive classifications, resulting in improvements across several diagnostic metrics. Although the effect is modest, these findings are consistent with prior related studies demonstrating the value of integrating imaging and structured clinical variables in diagnostic models [23] and support the concept that complementary clinical information can improve adjudication of diagnostically challenging cases without substantially altering overall model behavior. Although age, sex, and FVC are expected to correlate to some degree with CT-derived disease severity, the observed improvement of v2 over v1 across sensitivity, specificity, and diagnostic odds ratio indicates that these variables contribute incremental information beyond what CT imaging alone captures, rather than simply duplicating it; isolating the marginal contribution of each individual variable would require constructing additional model configurations outside the two versions specified under the current PCCP scope.

Performance was stable across CT manufacturers, acquisition protocols, slice thicknesses, and clinical sites for both versions, and the v2 update preserved that stability. Limited generalizability across imaging environments has been a recurring barrier to clinical deployment of machine-learning systems, so this consistency supports broader applicability. The fallback design reinforces this robustness: when age, sex, or FVC are unavailable, the system reverts to the validated CT-only v1 model, ensuring uninterrupted workflow and preserving case coverage. From a clinical implementation perspective, this fallback approach provides a practical and safe degradation pathway while enabling incremental adoption of multimodal inputs.

The clinical utility of Fibresolve v1 established in PUFAIR [3] was maintained, and modestly strengthened, in v2. The model has shown potential impact on indeterminate fibrotic-ILD cases that would otherwise undergo invasive sampling. In this “digital biopsy” subgroup, v2 achieved a non-invasive diagnostic yield of 56.2% and maintained high specificity (87.0%), comparable to or exceeding the 36.1% (95% CI 33.4–38.9%) yield reported for transbronchial biopsy [8]. Given that the median interval from initial assessment to final diagnosis in this cohort was 213 days [3], an earlier indication could shorten the diagnostic timeline and accelerate access to antifibrotic therapy, which slows lung-function decline in IPF [30,31] and across progressive fibrosing ILD more broadly [32].

The processes established under the PCCP framework enabled efficient incremental improvement of this FDA-regulated technology while preserving the safeguards appropriate to such a tool. By pre-specifying the scope of anticipated modifications, the methodology for implementing them, and the criteria for evaluating their impact, the framework allows model updates to proceed without a new regulatory review for each iteration—time-consuming and costly for developers and regulatory agencies alike—while constraining those updates to a defined envelope of permissible modification. The result is a structure oriented toward steady, controlled improvement: changes are advanced only when performance is maintained or improved, with continuous monitoring for performance drift and explicit attention to downside risk. Within this framework, head-to-head comparison of the modified model against the prior authorized version on a locked, adjudicated reference set of known cases provides a far more efficient validation pathway for algorithm improvements than commissioning a new clinical study for each revision. This matters because model performance continues to advance, as demonstrated here. Under prior regulatory paradigms, incorporating such improvements into an authorized clinical product could take years; the PCCP framework compresses that timeline substantially, allowing validated performance gains to reach clinical use far sooner—which, for a diagnostic tool, translates directly into earlier access to improved diagnostic accuracy for patients. For Fibresolve, this translates to upwards movements in sensitivity and specificity, translating to better diagnostic assessments for patients.

This study has several limitations. First, the analysis re-used the PUFAIR cohort and therefore represents a paired validation of the v2 update rather than a new distinct independent external validation study. Second, several subgroup analyses were limited by small sample sizes, which constrained the interpretation of stratum-level differences. Third, sensitivity remained limited among patients with normal lung function, suggesting that early or very mild disease remains a challenging diagnostic scenario. Lastly, ancillary clinical variables were not available for all patients; although the fallback architecture mitigates this limitation by reverting to the CT-only v1 model, some cases were evaluated using the original CT-only pathway.

## 5. Conclusions

In this paired validation study, Fibresolve v2 preserved the diagnostic performance and clinical utility established by v1 and demonstrated modest, directionally favorable improvements across the overall IPF cohort and multiple subgroups. The Fibresolve v2 algorithmic upgrade was implemented under the FDA Predetermined Change Control Plan (PCCP) framework by integrating age, sex, and FVC with CT imaging while retaining a validated fallback pathway to the CT-only v1 model, enabling an improved algorithm and setting the groundwork for additional improvements with time. Incorporation of multimodal data was shown to enhance performance of the system as well, highlighting the value of increased algorithm complexity and number of inputs where clinically relevant.

## Figures and Tables

**Table 1:** Overall diagnostic performance on the diagnostic thin-slice CT subset (N = 137; 40 IPF, 97 non-IPF).

Model	Sensitivity	Specificity	PPV	NPV	LR+	LR−	OR
Fibresolve v2	57.5 (42.2–71.5)	84.5 (76.0–90.4)	60.5 (44.7–74.4)	82.8 (74.2–89.0)	3.72 (2.18–6.36)	0.50 (0.35–0.73)	7.40 (3.21–17.03)
Fibresolve v1	55.0 (39.8–69.3)	82.5 (73.7–88.8)	56.4 (41.0–70.7)	81.6 (72.8–88.1)	3.14 (1.88–5.25)	0.55 (0.38–0.78)	5.75 (2.55–12.98)

v2 vs. v1: percent agreement 94.9%, Cohen κ = 0.87, McNemar exact *p* = 1.00. Abbreviations: CT, computed tomography; IPF, idiopathic pulmonary fibrosis; PPV, positive predictive value; NPV, negative predictive value; LR+, positive likelihood ratio; LR−, negative likelihood ratio; OR, diagnostic odds ratio; κ, Cohen’s kappa; v1, Fibresolve version 1; v2, Fibresolve version 2.

**Table 2:** Diagnostic performance in indeterminate thin-slice cases not confirmed as IPF by non-invasive criteria (N = 124; 32 IPF, 92 non-IPF).

Model	Sensitivity/ Yield	Specificity	PPV	NPV	LR+	LR−	OR
Fibresolve v2	56.2 (39.3–71.8)	87.0 (78.6–92.4)	60.0 (42.3–75.4)	85.1 (76.5–90.9)	4.31 (2.34–7.93)	0.50 (0.34–0.75)	8.57 (3.40–21.62)
Fibresolve v1	53.1 (36.4–69.1)	85.9 (77.3–91.6)	56.7 (39.2–72.6)	84.0 (75.3–90.1)	3.76 (2.06–6.85)	0.55 (0.37–0.80)	6.89 (2.78–17.09)

v2 vs. v1: percent agreement 95.2%, Cohen κ = 0.87, McNemar exact *p* = 1.00. Abbreviations: CT, computed tomography; IPF, idiopathic pulmonary fibrosis; PPV, positive predictive value; NPV, negative predictive value; LR+, positive likelihood ratio; LR−, negative likelihood ratio; OR, diagnostic odds ratio; κ, Cohen’s kappa; v1, Fibresolve version 1; v2, Fibresolve version 2.

**Table 3:** Overall diagnostic performance of Fibresolve v2 and v1 on the full dataset (N = 300; 83 IPF, 217 non-IPF). Values are percentages with 95% Wilson confidence intervals.

Model	Sensitivity	Specificity	PPV	NPV	LR+	LR−	OR
Fibresolve v2	44.6 (34.4–55.3)	88.0 (83.0–91.7)	58.7 (46.4–70.0)	80.6 (75.1–85.1)	3.72 (2.41–5.74)	0.63 (0.52–0.77)	5.91 (3.26–10.72)
Fibresolve v1	41.0 (31.0–51.7)	86.6 (81.5–90.5)	54.0 (41.8–65.7)	79.3 (73.7–84.0)	3.07 (2.00–4.69)	0.68 (0.57–0.82)	4.50 (2.50–8.09)

v2 vs. v1: percent agreement 96.7%, Cohen κ = 0.90, McNemar exact *p* = 1.00. Abbreviations: CI, confidence interval; IPF, idiopathic pulmonary fibrosis; PPV, positive predictive value; NPV, negative predictive value; LR+, positive likelihood ratio; LR−, negative likelihood ratio; OR, diagnostic odds ratio; κ, Cohen’s kappa; v1, Fibresolve version 1; v2, Fibresolve version 2.

**Table 4:** Sensitivity and specificity under sequential reference-standard exclusions, for the full dataset and the thin-slice CT subset.

Exclusion	N_remaining_	v2 Sens %, (95% CI)	v1 Sens %, (95% CI)	v2 Spec %, (95% CI)	v1 Spec %, (95% CI)
**Full dataset**					
Exclude UILD	246	44.6 (34.4–55.3)	41.0 (31.0–51.7)	90.2 (84.7–93.9)	90.2 (84.7–93.9)
Exclude CTD-ILD	287	44.6 (34.4–55.3)	41.0 (31.0–51.7)	87.7 (82.5–91.6)	86.8 (81.4–90.7)
Exclude CHP	250	44.6 (34.4–55.3)	41.0 (31.0–51.7)	86.2 (80.2–90.6)	84.4 (78.2–89.1)
Exclude all three	183	44.6 (34.4–55.3)	41.0 (31.0–51.7)	88.0 (80.2–93.0)	89.0 (81.4–93.7)
**Thin-slice subset**					
Exclude UILD	103	57.5 (42.2–71.5)	55.0 (39.8–69.3)	84.1 (73.2–91.1)	85.7 (75.0–92.3)
Exclude CTD-ILD	134	57.5 (42.2–71.5)	55.0 (39.8–69.3)	84.0 (75.3–90.1)	81.9 (72.9–88.4)
Exclude CHP	122	57.5 (42.2–71.5)	55.0 (39.8–69.3)	82.9 (73.4–89.5)	80.5 (70.6–87.6)
Exclude all three	85	57.5 (42.2–71.5)	55.0 (39.8–69.3)	80.0 (66.2–89.1)	82.2 (68.7–90.7)

Abbreviations: Sens, sensitivity; Spec, specificity; CI, confidence interval; CT, computed tomography; v1, Fibresolve version 1; v2, Fibresolve version 2; UILD, unclassifiable interstitial lung disease; CTD-ILD, connective tissue disease–associated interstitial lung disease; CHP, chronic hypersensitivity pneumonitis.

## Data Availability

The imaging and case-level clinical data analyzed in this study originate from the multinational registries and completed clinical trials described in the original PUFAIR study [3]; access to source data is controlled by the respective data-holding registries and partner institutions, not by the authors, and requests should be directed to those parties as outlined in [3]. The derived model outputs and performance analysis data generated for the present study are not publicly deposited but are available from the corresponding author upon reasonable request.

## Abbreviations

The following abbreviations are used in this manuscript:

IPF	idiopathic pulmonary fibrosis
ILD	interstitial lung disease
UILD	unclassifiable interstitial lung disease
CTD-ILD	connective tissue disease–associated ILD
CHP	chronic hypersensitivity pneumonitis
NSIP	nonspecific interstitial pneumonia
UIP	usual interstitial pneumonia
CT	computed tomography
CNN	convolutional neural network
ViT	vision transformer
FVC	forced vital capacity
ppFVC	percent-predicted forced vital capacity
FDA	U.S. Food and Drug Administration
PCCP	Predetermined Change Control Plan
SPS	SaMD Pre-Specifications
ACP	Algorithm Change Protocol
MDD	multidisciplinary discussion
PPV	positive predictive value
NPV	negative predictive value
LR+	positive likelihood ratio
LR−	negative likelihood ratio
OR	diagnostic odds ratio
CI	confidence interval

## Appendix A

**Table A1:** Baseline demographic and clinical characteristics of the cohort (full dataset, N = 300). The cohort is identical to that of the PUFAIR study [3].

Characteristic	% (n)
**Age**	
≤40	5.0 (15)
41–50	10.0 (30)
51–60	28.0 (84)
61–70	41.3 (124)
>70	15.7 (47)
**Sex**	
Female	49.7 (149)
Male	50.3 (151)
**Race**	
White	85.7 (257)
Black or African American	10.3 (31)
American Indian or Alaska Native	1.0 (3)
Multi-race	0.7 (2)
Asian	0.3 (1)
Unknown	2.0 (6)
**Ethnicity**	
Hispanic or Latino	9.0 (27)
Not Hispanic or Latino	88.7 (266)
Chooses not to disclose	2.3 (7)
**Smoking history**	
Ever	69.0 (207)
Never	31.0 (93)
**Lung function (ppFVC *)**	
<50%	19.7 (59)
50–75%	36.0 (108)
>75%	34.7 (104)
**Surgical pathology**	
Recorded	94.7 (284)
None recorded	5.3 (16)

* ppFVC, percent-predicted forced vital capacity; data not available for 29 patients.

**Table A2:** Cohort breakdown by final multidisciplinary-discussion diagnosis (full dataset, N = 300).

Final Diagnosis	% (n)
IPF	27.7 (83)
Not IPF (all)	72.3 (217)
Unclassifiable ILD (UILD)	18.0 (54)
Chronic hypersensitivity pneumonitis	16.7 (50)
Nonspecific interstitial pneumonia	10.7 (32)
Cryptogenic organizing pneumonia	7.7 (23)
Connective-tissue-disease-associated ILD	4.3 (13)
Desquamative interstitial pneumonia	3.0 (9)
Eosinophilic granulomatosis with polyangiitis	3.0 (9)
Sarcoidosis	2.7 (8)
No ILD	1.3 (4)
Berylliosis	1.3 (4)
Respiratory bronchiolitis–ILD	1.0 (3)
Chronic eosinophilic pneumonia	0.3 (1)
Lymphocytic interstitial pneumonia	0.3 (1)

Abbreviations: IPF, idiopathic pulmonary fibrosis; ILD, interstitial lung disease; UILD, unclassifiable interstitial lung disease.
